# Isolation and Characterization of *Pseudomonas* spp. Strains That Efficiently Decompose Sodium Dodecyl Sulfate

**DOI:** 10.3389/fmicb.2017.01872

**Published:** 2017-11-07

**Authors:** Ewa M. Furmanczyk, Michal A. Kaminski, Grzegorz Spolnik, Maciej Sojka, Witold Danikiewicz, Andrzej Dziembowski, Leszek Lipinski, Adam Sobczak

**Affiliations:** ^1^Institute of Biochemistry and Biophysics, Polish Academy of Sciences, Warsaw, Poland; ^2^Institute of Organic Chemistry, Polish Academy of Sciences, Warsaw, Poland; ^3^Institute of Genetics and Biotechnology, Faculty of Biology, University of Warsaw, Warsaw, Poland

**Keywords:** biodegradation, xenobiotics, sodium dodecyl sulfate, alkyl sulfatase, *Pseudomonas* sp., surface flow constructed wetland, SPME-GC-MS, biodiversity

## Abstract

Due to their particular properties, detergents are widely used in household cleaning products, cosmetics, pharmaceuticals, and in agriculture as adjuvants tailoring the features of pesticides or other crop protection agents. The continuously growing use of these various products means that water soluble detergents have become one of the most problematic groups of pollutants for the aquatic and terrestrial environments. Thus it is important to identify bacteria having the ability to survive in the presence of large quantities of detergent and efficiently decompose it to non-surface active compounds. In this study, we used peaty soil sampled from a surface flow constructed wetland in a wastewater treatment plant to isolate bacteria that degrade sodium dodecyl sulfate (SDS). We identified and initially characterized 36 *Pseudomonas* spp. strains that varied significantly in their ability to use SDS as their sole carbon source. Five isolates having the closest taxonomic relationship to the *Pseudomonas jessenii* subgroup appeared to be the most efficient SDS degraders, decomposing from 80 to 100% of the SDS present in an initial concentration 1 g/L in less than 24 h. These isolates exhibited significant differences in degree of SDS degradation, their resistance to high detergent concentration (ranging from 2.5 g/L up to 10 g/L or higher), and in chemotaxis toward SDS on a plate test. Mass spectrometry revealed several SDS degradation products, 1-dodecanol being dominant; however, traces of dodecanal, 2-dodecanol, and 3-dodecanol were also observed, but no dodecanoic acid. Native polyacrylamide gel electrophoresis zymography revealed that all of the selected isolates possessed alkylsulfatase-like activity. Three isolates, AP3_10, AP3_20, and AP3_22, showed a single band on native PAGE zymography, that could be the result of alkylsulfatase activity, whereas for isolates AP3_16 and AP3_19 two bands were observed. Moreover, the AP3_22 strain exhibited a band in presence of both glucose and SDS, whereas in other isolates, the band was visible solely in presence of detergent in the culture medium. This suggests that these microorganisms isolated from peaty soil exhibit exceptional capabilities to survive in, and break down SDS, and they should be considered as a valuable source of biotechnological tools for future bioremediation and industrial applications.

## Introduction

Surfactants are amphiphilic compounds with both hydrophilic and hydrophobic parts. This allows them to accumulate at the interfaces between air and water, or water and oil and lower the surface tension. According to their charge in aqueous solutions, surfactants can be grouped into anionic, non-ionic, cationic, or amphoteric classes (Im et al., 2008). The low price and beneficial properties of anionic surfactants make them popular additives to a wide range of products like: cosmetics, pharmaceuticals, household and industrial cleaning products, and in agriculture as adjuvants improving spraying properties and pesticide penetration.

The extensive application of surfactants in household and agricultural products results in an accumulation of these compounds in aquatic and terrestrial environments, giving rise to toxic effects on living organisms. Anionic detergents such as SDS are known to have bacteriostatic or even bactericidal properties and inhibit the growth of some nitrogen-fixing cyanobacteria, algae, crustaceans (Lechuga et al., 2016), and also fishes (Sandbacka et al., 2000). The features underlying their accumulation in living organisms and toxicity are the amphoteric properties of these detergents, which promote interactions with intracellular components by means of both electrostatic (by the negatively charged head) or hydrophobic (by the hydrophobic part) forces (Cserhati et al., 2002).

Problems caused by large amounts of surfactants are clearly visible in sewage treatment plants, where detergents present in the wastewater negatively influence the physicochemical and biological processes employed in water purification. On the physicochemical level, the decreased surface tension of the liquid causes a deterioration in the flocculation and sedimentation of small particles by stabilizing their colloidal suspension. On the biological level, anionic surfactants affect the functioning of the sludge microbial consortium on several levels, having a negative effect on its biodiversity and a pivotal role in the decomposition of numerous xenobiotics. Among the numerous possible mechanisms underlying the negative influence of anionic surfactants on living organisms, several have already been determined (Ivanković and Hrenović, 2010): (I) absorption of detergents at the surface of activated sludge flocs, triggering bacterial cell lysis; (II) interactions with proteins, causing disruption and conformational changes to their tertiary structure; and (III) binding at enzymes’ active sites or substrate-binding pockets, influencing chemical reactions. In consequence, surface-active xenobiotics decrease the metabolism of microorganisms, which lose the activity of certain groups of enzymes, and thus change the degradation profile of some compounds (e.g., carbohydrates) (Eerlingen et al., 1994). Overall, high surfactant concentrations in wastewater decrease biodiversity and the metabolic processes conducted within active sludge consortia, making the whole water purification process inefficient and costly (Zangeneh et al., 2014).

Despite their known toxicity, the use of anionic detergents is often proposed for the bioremediation of hydrocarbon-contaminated soils or water, due to their ability to increase the water solubility and bioavailability of many hydrophobic xenobiotics. In this context, SDS is one of the most popular detergents proposed for soil bioremediation (Yu et al., 2007; Zhou and Zhu, 2008; Moldes et al., 2013). However, bacterially augmented bioremediation in the presence of SDS is not an easy process, requiring the use of several types of microorganisms: (a) xenobiotic decomposers applied in the first step of bioremediation, resistant to the presence of anionic detergent, and (b) efficient detergent degraders used in the second step.

There are various reports describing the isolation of bacteria having the capacity to degrade surfactants, including a wide variety of *Pseudomonas* strains (Klebensberger et al., 2006; Chaturvedi and Kumar, 2011a; Yeldho et al., 2011; John et al., 2015), *Klebsiella oxytoca* (Shukor et al., 2009; Masdor et al., 2015), *Enterobacter* sp. strain NENI-13 (Rahman et al., 2016), and finally microbial consortia composed of *Acinetobacter calcoaceticus* and *Pantoea agglomerans* (Abboud et al., 2007). However, these microorganisms were selected from environments highly contaminated solely with detergents with no other xenobiotics present.

In this study, we sampled peaty soil coming from the root zone of a surface flow constructed wetland of a wastewater treatment plant operated by a pesticide packing company, having excellent sewage purification properties. As detergents are widely used as adjuvants, tailoring the properties of crop protection products to the end-user’s needs, we assumed that microorganisms inhabiting this soil sample should have the ability to efficiently decompose detergents, combined with high resistance to such toxic xenobiotics. We found it intriguing to compare the microbial biodiversity of the analyzed peaty soil sample and the taxonomy of the isolated microorganisms having the desired metabolic properties. Such a comparison could be helpful in elucidating which culturable taxonomic groups could be considered as responsible for detergent decomposition in the soil sampled from the root zone. Finally, we provided initial biochemical characterization of the most promising isolates, those that exhibited desirable features, such as efficient SDS decomposition combined with resistance to substantial detergent concentrations.

## Materials and Methods

### Soil Sample Collection

A soil sample was collected from a subsurface flow constructed wetland of a wastewater treatment plant operated by Agropak s.j. (Jaworzno City, Poland), a Polish pesticide producer, where large amounts of peaty soil are used as a biologically active filter. The samples were taken from a depth of 5–30 cm at four independent sites (with 50 cm distances between) forming a parallel line 5 m from the basin collecting the pesticide-contaminated water. The subsamples were combined obtaining approximately 3 kg of soil which were thoroughly mixed to homogenize and sieved with 1/4” and 1/16” sieves. For DNA isolation, homogenized soil was subsampled in and frozen with dry ice in the field, then stored at -80°C. For bacterial isolation, soil samples were placed in plastic bags and stored at 8°C.

### Media Preparation

Lysogeny broth (LB) medium (per liter: 10 g tryptone; 5 g yeast extract; 5 g NaCl) (Sambrook and Russel, 2001), 10 times diluted LB medium (0.1X LB), basal medium [per liter: 3.5 g KH_2_PO_4_; 1.5 g K_2_HPO_4_; 0.25 g (NH_4_)_2_SO_4_; 0.5 g NaCl; 0.14 g MgSO_4_; 0.15 g MgCl_2_.6H_2_O] (Shahbazi et al., 2013), or minimal medium [per liter: 0.5 g Na_2_HPO_4_; 0.5 g KH_2_PO_4_; 0.25 g (NH_4_)_2_SO_4_] were used during this study. The soil extract was prepared by autoclaving of 500 g of air-dried garden soil mixed with 1 L of tap water for 1 h at 121°C. Solid particles were centrifuged for at least 10 min, and the resulting supernatant was used for further experiments. Solid media were obtained by the addition of 1.5% agar. SDS (1 g/L or 2 g/L) was used as a carbon source in the minimal media. The 20% (w/v) SDS (Sigma–Aldrich) and 20% (w/v) glucose (POCH) stock solutions were prepared in ultrapure water and sterilized by filtration.

### Isolation of SDS-Degrading Bacteria

Ten gram of the collected soil stored at 8°C was placed into the sterile container of a Waring blender, and 100 mL of 0.9% NaCl was added. The sample was homogenized by three blending cycles at maximum speed for 1 min, with intermittent cooling on ice for 1 min. After 5 min of sedimentation, the liquid fraction was transferred into a sterile beaker. Another 100 mL of 0.9% NaCl was then added to the soil sludge, and the whole procedure was repeated three times in total. All the fractions were pooled together and centrifuged at 5,000 *g* for 10 min at 4°C. Several dilutions of the obtained pellet were plated on solid soil extracts plates. After 7 days of incubation at 23°C, colonies with unique morphologies were transferred into 96-well plates containing soil extract supplemented with 15% glycerol and stored at -80°C.

Isolated microorganisms were replicated on solid basal medium plates with SDS (2 g/L) as the carbon source. In the basal medium SDS precipitates make this medium suitable for observations, as a clear halo surrounding growing bacterial colonies forms, indicating detergent degradation. To shorten the time of colony growth and halo formation, plates were incubated at an increased temperature of 30°C for 3–4 days. Isolates with clear halos around the colonies were considered to have SDS degrading features. They were then verified to be pure by two independent streaking passages on the same medium, and finally selected for further SDS biodegradation study.

### Preliminary SDS Biodegradation Test: the Colorimetric Assay

The selected isolates were pre-cultured overnight in liquid 0.1X LB medium unsupplemented with detergent at 30°C with orbital agitation (140 rpm). Minimal medium supplemented with 0.1% SDS (1 g/L) was inoculated to OD_600_ = 0.05–0.15 with the overnight culture, which had been washed with minimal medium without any carbon source. The detergent concentration was measured by colorimetric assay at 0, 2, 4, 6, 8, and 24 h after the beginning of the experiment. The minimal medium was used in liquid cultures for colorimetric assays because there was no SDS precipitation observed under this condition.

The SDS concentration was determined in a 96-well plate using Stains-All reagent (Sigma–Aldrich) as described elsewhere (Rusconi et al., 2001) with minor modifications. Stains-All stock solution (1 mg/mL) was prepared in isopropanol:water (50:50). The working solution (WS) consisted of 1 mL of the stock diluted in 18 mL of water and 1 mL of formamide. Samples from each time points were diluted 10 times, 8 μL of diluted sample were dispensed into the well, supplemented with 100 μL of water and 100 μL of the Stains-All working solution, and finally the absorbance (at 438 nm) was measured after 10 min of incubation at RT. The measurements were carried out in triplicate. Results of colorimetric assay were confirmed also by mass spectrometry.

### Isolation and Purification of Soil Total DNA

The metagenomic DNA isolation was done in triplicate. Five grams of soil sample was resuspended in 4 mL Zhou buffer (Zhou et al., 1996); 2 g of 0.5 mm zirconia/silica beads were added, and the mixture was vortexed for 15 s at maximum rpm. After centrifugation, the supernatant was transferred to a new tube and 4 mL of fresh Zhou buffer was added to the soil. The procedure was repeated two times, and the supernatants were combined together into fraction A. Six mL of Zhou buffer was added to the remaining soil sediment and mixed (fraction B). One hundred μL of lysozyme (100 mg/mL), 34 μL of achromopeptidase (100 kU/mL), and 25 μL RNase A (10 mg/mL) were added to each fraction, and they were incubated for 1 h in a water bath at 37°C. After this time, 25 μL of proteinase K (40 mg/mL) was added, and the fractions were incubated for 30 min at 37°C. Next 0.75 mL of 20% SDS was added, and the suspension was incubated for 2 h in a water bath at 55°C with mixing by inversion at 15 min intervals. After this time, each sample was chilled on ice for 2 min, centrifuged (20 min, RT, 8,000 rpm), and the resulting supernatant was extracted twice with equal volumes of chloroform. The DNA was then precipitated overnight in the presence of 10% PEG 8000 at 4°C. The DNA pellet from each fraction was centrifuged (45 min, 4°C, 10,000 *g*), washed twice with 70% ethanol and resuspended in 200 μL of water.

The whole community DNA was purified using Q Sepharose (Sigma–Aldrich). Briefly, the resin was first washed with TE buffer, and then 200 μL samples of isolated DNA from each fraction were combined with 100 μL of the resin. The solution was mixed for 15 min by inversion at RT. After a short centrifugation (15 s, 800 rpm), the supernatants were transferred to new tubes. The procedure was repeated once for DNA from fractions A and six times for DNA from fractions B. The fractions were combined in an equal concentration ratio, and 100 μL of this solution was purified using AMPureXP (1:1 ratio) (Beckman Coulter) and resuspended in 50 μL of water.

### Metagenomic Library Preparation and Sequencing

Two types of libraries were constructed using the amplified V3–V4 16S rRNA region (464 bp): the first one, AP3MET, representing the metagenomic diversity of the soil sample; and the second, AP3BAC, representing the biodiversity of the preselected SDS-degrading bacterial isolates. For the AP3MET library, 100 ng of the purified metagenomic DNA was used as a template. For the AP3BAC library, the amplicons for individual isolates were amplified separately using colony PCR. A single colony of each selected isolate, cultured on basal medium with SDS as a sole carbon source, was resuspended in 20 μL of PCR lysis solution (50 mM NaOH; 0.25% SDS) and boiled at 99°C for 10 min. The samples were then placed on ice, and 180 μL of cold sterile water was added. These solutions were used as templates for PCR. In both cases, PCR was performed in a TProfessional Basic Thermocycler (Biometra), using Phusion polymerase (0.02 U/μl, Thermo; supplied with 1x HF-buffer), 0.2 mM dNTP mixture, and primers specific to V3–V4 region of 16S rRNA gene S-D-Bact-0341-b-S-17 (CCTACGGGNGGCWGCAG) and S-D-Bact-0785-a-A-21 (GACTACHVGGGTATCTAATCC) (Klindworth et al., 2013), – 0.4 μM each, under the following conditions: one cycle at 96°C for 2 min, 21 or 25 cycles (for bacterial or environmental DNA) at 96°C for 30 s, 54°C for 50 s, and 72°C for 20 s with final extension at 72°C for 5 min. The amplified DNA fragments were purified using AMPureXP beads (1:1 ratio) according to the manufacturer’s instructions. The products from each of the preselected SDS-degrading isolates (for the AP3BAC library) were purified separately and then pooled together in equal molar ratios. Approximately 250 ng of purified PCR product was used for each library preparation using the KAPA HTP Library Preparation Kit for Illumina platforms according to the manufacturer’s protocol, except that the final library amplification was omitted. The libraries were verified using a 2100 Bioanalyzer (Agilent) High-Sensitivity DNA assay and a KAPA Library Quantification Kit for Illumina. Pair-end sequencing was performed using an Illumina MiSeq (MiSeq Reagent Kit v3, 600 cycles) with a read length of 2 × 300 bp.

### Bioinformatic Analysis of High-Throughput Amplicon Data

The obtained reads for each library were filtered to quality scores (Q30) and then merged using FLASH software (Magoč and Salzberg, 2011). The adaptor and primer sequences were deleted using the Cutadapt script (Martin, 2011). Merged sequences with at least 400 bp were used in analysis, with various scripts implemented in QIIME (v 1.9) (Caporaso et al., 2010). Firstly, they were clustered into operational taxonomic units (OTU) with an identity threshold of 97% using ULUST (Edgar, 2010). Representative sequences for each OTU were used to assign taxonomy using the RDP classifier (Wang et al., 2007) against the SILVA (Quast et al., 2013) database (release 128) and to build an OTU table. Amplicon sequences were deposited in the NCBI’s SRA database (BioProject PRJNA361553).

### Bacteria Identification via 16S rRNA Gene Analysis and Phylogenetic Tree Construction

The 16S rRNA gene (approximately 1,500 bp) of the top five degraders was amplified by colony PCR using the following universal primer pair: 27F (AGAGTTTGATCCTGGCTCAG) and 1492R (GGTTACCTTGTTACGACTT) (Lane, 1991). A single colony of each selected isolate grown on basal minimal medium with SDS as a carbon source was resuspended in 20 μL of PCR lysis solution (50 mM NaOH; 0.25% SDS) and boiled at 99°C for 10 min. The samples were then placed on ice, and 180 μL of cold sterile water was added. These solutions were used as templates. The PCR was performed in 50 μL using Phusion High-Fidelity DNA Polymerase (Thermo; supplied HF Buffer and other components as mentioned previously). The PCR involved: one cycle at 99°C for 5 min; 10 cycles at 99°C for 30 s, 60°C for 30 s, and 72°C for 45 s; and 20 cycles at 99°C for 30 s, 50°C for 30 s, and 72°C for 45 s with final extension at 72°C for 5 min. The PCR products (approximately 1,500 bp) of 16S rRNA genes were purified using AMPureXP with a 0.8:1 ratio, then cloned into a pCR^TM^Blunt II-TOPO^®^ vector (Thermo Fisher) according to the manufacturer’s protocol, and transformed to *Escherichia coli* (Sambrook and Russel, 2001). The plasmids were isolated using a Plasmid Mini kit (A&A Biotechnology) and the inserts were sequenced with Sanger using universal M13 Forward (GTAAAACGACGGCCAG) and M13 Reverse (CAGGAAACAGCTATGAC) primers (Thermo Fisher). 16S rRNA partial gene sequences were deposited at the NCBI (GenBank accession numbers: KY462011-KY462014 and MF554631). The sequences were compared to the EzBioCloud database (Yoon et al., 2017).

The sequences were aligned to each other using ClustalW (Larkin et al., 2007). 16S rRNA gene sequences of the five selected SDS-degrading isolates and 27 representative sequences of reference type strains from the NCBI database were used to construct a phylogenetic tree. Multiple sequence alignment to identify the maximum identical gene fragment was carried out using ClustalW (Larkin et al., 2007). A maximum likelihood tree was constructed in MEGA 6.0 (Tamura et al., 2013) with 1,000 bootstrap replicates using Tamura-Nei model and default settings.

### Characterization of the SDS-Degrading Ability by the Selected Isolates

#### Effect of SDS Concentration on Bacterial Growth

The selected isolates were pre-cultured overnight in LB medium as mentioned before, washed two times with minimal medium without any carbon source, and then diluted to OD_600_ = 0.15 in minimal medium with different SDS concentrations (0.5–50 g/L) as the sole carbon source. The isolates were cultured in triplicate in 48-deep well plates. The optical density of the cultures was monitored after 24 h of incubation. Statistical analysis and interpretation of obtained data was made using the R package version 3.5.0. The Kruskal–Wallis with the Dunn’s *post hoc* tests were used to test differences between initial and final optical density of cultures, with significance set at a probability smaller than 0.05.

#### Drop Plate Assay for Chemotaxis

Overnight LB-pre-cultures of the selected isolates were used to inoculate fresh 50 mL LB medium (1:50, v/v). After 2 h of incubation at 30°C, cells were harvested by centrifugation at 8,000 rpm for 15 min, washed twice with 0.9% NaCl, resuspended to a final concentration OD_600_ = 0.1 in minimal medium containing 0.45% bacto agar and poured into 30 mm petri plates. Next five μL of 20% SDS or 20% glucose were dropped into the center of the petri plate, and the chemotactic response was observed after 8 h of incubation at 30°C.

#### LC-MS/MS Quantitation of SDS

For chemical analysis of SDS degradation, the selected isolates were pre-cultured in LB medium at 30°C with agitation (140 rpm). The cultures were then diluted to OD_600_ 0.1–0.15 in 50 mL of minimal medium supplemented with 0.1% SDS as the sole carbon source and cultured as mentioned above. After 24 h of growth, cells were centrifuged at 10,000 rpm for 15 min, and the concentration of SDS was measured using a Prominence LC-20 HPLC (Shimadzu) coupled with a 4000 QTRAP tandem mass spectrometer (SCIEX) equipped with an electrospray ion source. The separation was performed using a 4.6 mm × 250 mm XDB-C8 (5 μm) column (Agilent). A gradient flow was used for all measurements. Solvent A consisted of 5 mM ammonium acetate in water, while the solvent B was acetonitrile (both HPLC grade). The gradient ran from 50 to 100% of solvent B in 5 min, 100% of solvent B was maintained for 2 min, and was then returned to the initial conditions. For quantitative analysis, multiple reaction monitoring (MRM) experiments were carried out in negative ion mode. The *m/z* 265/80 and 265/97 precursor/product ion pairs were used as quantitative and confirmative ions, respectively. Zero air was used as the nebulizer gas, and nitrogen as the curtain gas. The tip voltage was kept at 4.5 kV. The compound dependent declustering potential and collision energy values were set, and the dwell time was 20 ms. Standard solutions were prepared by dissolving the SDS standard in water in a range from 30 to 600 ng/mL. All supernatant samples were diluted by 50 times twice, to reach the optimum concentration for the applied method.

#### SPME-GC-MS Analysis

The analysis of potential SDS metabolites in supernatants was performed using the solid-phase microextraction technique by suspending a 100 μm, 24 ga PDMS fiber (Supelco) above 2 mL of the supernatant sample placed in a 20 mL closed headspace vial. The sampling process was carried out for 30 min for each sample, and immediately afterward the fiber was inserted into the injector, and heated to 260°C to start the analysis. GC-MS analyses were performed on an Agilent 7890A gas chromatograph coupled with an Agilent 5975C mass spectrometer equipped with an EI (electron ionization) source and a single quadrupole mass analyser. Separation was performed on an Agilent HP-5ms column (30 m × 250 μm × 0.25 μm). The carrier gas was helium, and the flow rate was 1 mL/min. The temperature program was 100°C for 2 min, then 15°/min to 300°C, and 300°C for 10 min.

#### Crude Cell Extracts Preparation for Native PAGE Zymography

Bacterial cells were precultured overnight in LB medium, diluted to OD_600_ = 0.15 in 100 ml of minimal medium supplemented with 1 g/L SDS or glucose as the carbon source, and incubated for 20 h at 30°C. Cells were harvested by centrifugation at 10,000 *g* for 15 min at RT. Cell pellets were resuspended in lysis buffer (50 mM HEPES, pH 7.5, 300 mM NaCl, 20 mM imidazole, 50 μM PMSF, 10 mM β-mercaptoethanol, 0.1% Tween 20, 10% glycerol, and lysozyme) and disrupted by sonication in a Diagenode sonication system in a cooled water bath (4°C) at high power (300 W) for 30 cycles of 30 s on and 30 s off. Cell debris was removed by centrifugation at 14,000 *g* for 30 min at 4°C and the supernatants were stored at -20°C.

To determine the sulfatase activity, the crude cell extracts (60 μg of protein) were separated using 8% native polyacrylamide gel (Sambrook and Russel, 2001). The electrophoresis was carried out at 100 V at 4°C in 0.378 M Tris-glycine buffer (pH 8.3). After washing in ultrapure water, the gel was incubated in a developing solution containing 20 mM SDS in 0.1 M Tris-Cl (pH 7.5) at 30°C. Active alkyl sulfatases was visualized by the formation of white bands of insoluble alcohol.

## Results

### Isolation of SDS-Degrading Microorganisms

**FIGURE 1:**
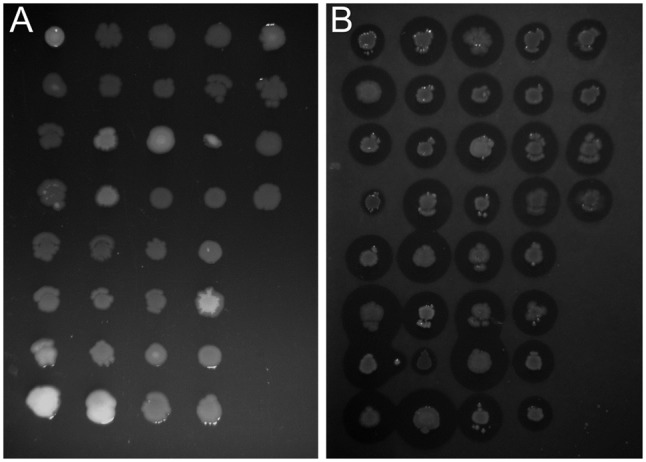
Identification of bacteria potentially degrading sodium dodecyl sulfate (SDS). Comparison of growth profile of selected strains cultured on solidified 0.1X LB **(A)** or basal medium supplemented with 0.2% SDS **(B)**. Differences in clear zone diameter are observed for each strain cultured on SDS-containing medium indicate a variety of degree of detergent metabolism.

**FIGURE 2:**
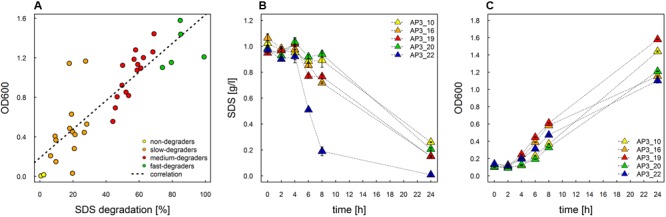
Study of SDS degradation of selected isolates. **(A)** Correlation of growth rate and SDS degradation (decrease in substrate concentration [%]) after 24 h of incubation plotted for the 36 selected strains. The dotted line represents correlation. **(B)** Time course study of degree of SDS degradation of the top five selected strains. **(C)** Time course study of growth rate for the five most effective degraders selected during the experiment. The values are means of the three replicates, and the error bars indicate the standard deviations.

In this study we used the peaty soil from biological wastewater treatment plant regularly exposed to pesticides and detergents as a source of unknown SDS-degrading microorganisms. The isolation procedure based on the ability to grow on the solid soil extract under laboratory conditions resulted in the selection of 238 morphologically distinctive colonies. Their ability to survive and decompose SDS was assessed on solid basal medium supplemented with SDS (2 g/L) as the sole carbon source. For 36 isolates, a clear halo surrounding the colony was observed, which was interpreted as the ability to decompose SDS (compare **Figures 1A,B**). This assumption was further confirmed in liquid cultures by a SDS-measuring colorimetric assay (see **Figure 2**, and for details Supplementary Table S1). Two isolates did not adapt to liquid culture, showing minor growth with no SDS decomposition observed after 24 h of incubation. In the remaining 34 cases, degradation of SDS was observed as a decrease from the initial detergent concentration in liquid culture measured in percent ranged from 6.4 to 99.2% (**Figure 2A**). Based on the degree of SDS degradation, the isolates were arranged into four metabolic classes reflecting their SDS-degradation ability: non-degraders (2 isolates), slow-degraders (14 isolates with an SDS degree of degradation below 30%), medium degraders (15 isolates with a degree of SDS degradation between 31 and 70%), and fast degraders (5 isolates with the degree of SDS degradation exceeding 70%). The most promising isolate, AP3_22, utilized approximately 50 and 81% of SDS within 6 and 8 h of the experiment, respectively (**Figure 2B**). After 24 h, the fast degrading isolates were able to degrade: 74.1% – AP3_10; 84.6% – AP3_16; 85.1% – AP3_19, 79.4% – AP3_20, and 99.2% – AP3_22 of SDS (from the initial 1 g/L). This experiment showed a strong positive correlation (correlation coefficient *r* = 0.86) between the degree of SDS degradation and the optical density of the isolates liquid cultures (**Figure 2A**). Analysis of OD_600_ changes for isolates cultured in liquid media revealed differences in bacterial behavior in response to detergent exposure. For 23 isolates, a decrease in optical density was observed during the lag phase, usually lasting 2 h, which could be interpreted as an adaptation phase (Supplementary Table S1). In contrast, another 11 isolates were characterized with a very short lag phase and continuous culture growth during the whole experiment. From the fast degraders group isolate AP3_19 was able to actively grow almost from the beginning of the experiment having the shortest adaptation phase (**Figure 2C**). However, in case of this isolate the most efficient growth did not translate to the highest degree of SDS degradation (**Figure 2B**), as the AP3_19 isolate turned to be second the most efficient degrader. Isolates AP3_10 and AP3_20 showed slower initial growth, nonetheless, it did not affect their final optical density (**Figure 2C**). Isolates AP3_16 and AP3_22 were characterized with very similar growth patterns (with very short lag phase) but they differ significantly in SDS degradation (compare **Figures 2B,C**).

### Biodiversity of the Soil Sample and Taxonomic Analysis of the Isolated SDS-Degrading Bacteria

#### Biodiversity of the Soil Sample

Next we analyzed the taxonomy of the isolated culturable SDS-degrading microorganisms in context of the taxonomic biodiversity of soil sample of their origin. For the taxonomic biodiversity of soil sample, whole microbial community DNA was extracted, and amplicons of the V3–V4 region of the 16S rRNA gene were sequenced on an Illumina MiSeq platform, resulting in 252,124 paired reads. After quality filtering, read merging, adaptor and primer trimming, and length filtering, 206,771 sequences were obtained and used in a biodiversity analysis performed with QIIME.

A total of 99.94% of the sequences were assigned to various taxonomic levels inside domain Bacteria, grouped in 45 different phyla, while 0.06% sequences remained unclassified.

**FIGURE 3:**
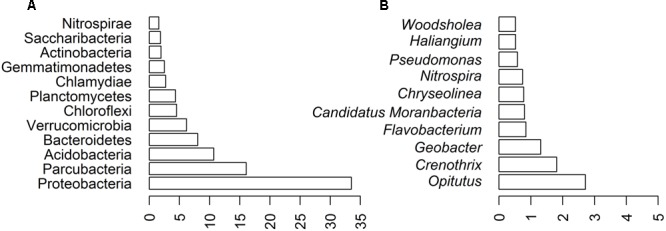
Biodiversity of AP3 soil sample based on bioinformatics analysis of deep sequencing of AP3MET library. Community composition at different taxonomic ranks: **(A)** phylum level. **(B)** 10 most represented genera in the soil sample.

The most abundant 12 phyla (>1% sequences) comprised 94.02% of the AP3 community (**Figure 3A**), within which 33.52% of the sequences belonged to the *Proteobacteria* phylum. The second most numerous phylum was *Parcubacteria* (16.04%), also known as *OD1*, gathering non-culturable bacteria widespread in various anoxic environments (Nelson and Stegen, 2015). Other common phyla identified in the AP3 soil sample were *Acidobacteria* (10.70%), *Bacteroidetes* (8.04%), and *Verrucomicrobia* (6.18%). *Planctomycetes, Chloroflexi, Chlamydiae*, and *Gemmatimonadetes* were less prevalent phyla.

Further analysis showed that the *Proteobacteria* phylum was mainly composed of four classes: *Alphaproteobacteria* (11.07%) with *Rhizobiales* (5.95%) as the main order, *Betaproteobacteria* (8.85%) with *Nitrosomonadales* (5.36%) as the main order, *Gammaproteobacteria* (7.26%) with *Methylococcales* (3.04%) as the main order, and *Deltaproteobacteria* (6.09%) with *Myxococcales* (2.01%) as the main order. Detailed taxonomic representation at the aforementioned taxonomic levels is presented in the Supplementary Figure S1. Fifty-three bacterial genera were present in at least 0.1% relative abundance of sequences. *Opitutus* (2.72%) was the most abundant genus. Also *Crenothrix* (1.81%) and *Geobacter* (1.31%), belonging to the *Proteobacteria* phylum, were among the most abundant genera representing the bacterial community. The 10 most represented genera are shown on **Figure 3B**.

#### Taxonomic Analysis of Isolated Bacteria

In next step, taxonomic classification of the 36 promising isolates was confirmed by sequencing of the amplicons comprising V3–V4 region of the 16S rRNA gene. From 95,564 paired reads for the bacterial V3–V4 library (AP3BAC) after quality filtering (Q30), read merging, adaptor and primer trimming, and length filtering, 60,063 sequences were employed in the biodiversity analysis with QIIME. A total of 97.45% of the sequences were assigned to the Bacteria domain, grouped in only one phylum – *Proteobacteria*. The other 2.55% sequences remained unclassified. All of the sequences with assigned taxonomy were in the genus *Pseudomonas* (97.45%) from the *Gammaproteobacteria* class. Despite the fact that the AP3 soil sample was extremely reach in terms of bacterial diversity, and only 0.58% of the sequences from whole sample DNA sequencing were assigned to the *Pseudomonas* genus, the isolated SDS-degrading microorganisms all belonged to this particular group.

### Direct Taxonomic Identification of Isolates of Fast Degraders

**FIGURE 4:**
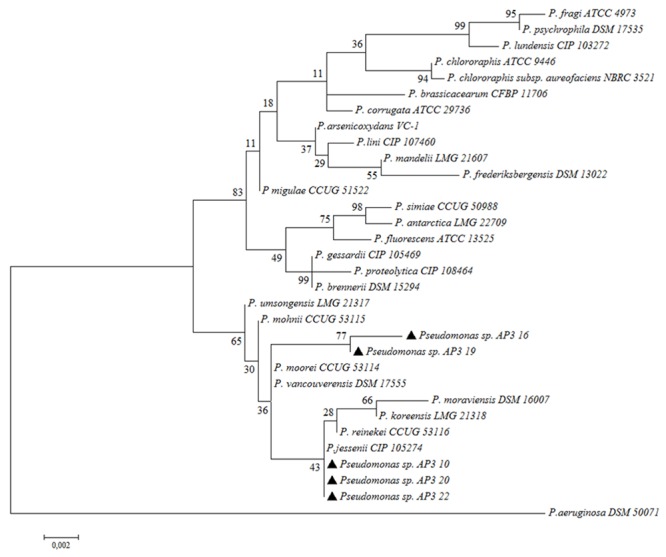
Phylogenetic tree of selected isolates among chosen representatives of Pseudomonas. The phylogenetic tree was computed by Maximum Likelihood with 1,000 bootstrap replicates under an Tamura-Nei model. Examined isolates are marked with black triangles.

In the last step, detailed classification by Sanger sequencing of cloned 16S rRNA gene amplicons (approximately 1500 bp) for the five fast-degrading isolates was determined. The alignment of the obtained sequences indicated that the isolates were highly similar to each other but not identical (99.93% identity for the most similar isolate pair – AP3_10 and AP3_22). The comparison of these sequences to the EzBioCloud database revealed that the four of the fast-degrader isolates showed the highest identity (AP3_10 – 99.79%, AP3_16 – 99.24%, AP3_20 – 99.86%, and AP3_22 – 99.79%) to *Pseudomonas jessenii* CIP 105274. However, the AP3_19 isolate shared the highest identity (99.45%) with *P. mohnii* Ipa-2. **Figure 4** shows that the entire SDS-degrading group falls within a clade. AP3_10, AP3_16 and AP3_22 isolates clustered with *P. jessenii* CIP 1052274, *P. reinekei* CCUG 53116, *P. koreensis* LMG 21318, and *P. moraviensis* DSM 16007, while AP3_16, and AP3_19 were sister taxa that clustered together in a group that included the previously mentioned clade, plus *P. moorei* CCUG 53114, and *P. vancouverensis* DSM 17555. Low bootstrap values cast doubt on the precise relationships among these taxa, and the entire group of interest may also include *P. umsongensis* LMG 21317 and *P. mohnii* CCUG 53115. However, it would be an unlikely coincidence for all of these SDS-degrading bacteria to cluster within the same clade, apart from other *Pseudomonas* bacteria. Above results for the first time indicate that microorganisms from *P. jessenii* subgroup inhabiting terrestrial environment could be involved in SDS degradation.

### Characterization of Fast SDS-Degraders

The most promising isolates belonging to the fast degraders group were examined for: (I) bacterial growth in a range of SDS concentrations, (II) chemotactic response toward SDS, (III) LC-MS/MS analysis of SDS degradation and the identification of the degradation products by SPME-GC-MS, and (IV) Native PAGE zymography to confirm the presence of enzymes involved in SDS degradation.

#### Effect of SDS Concentration on the Fast Degraders’ Growth

**FIGURE 5:**
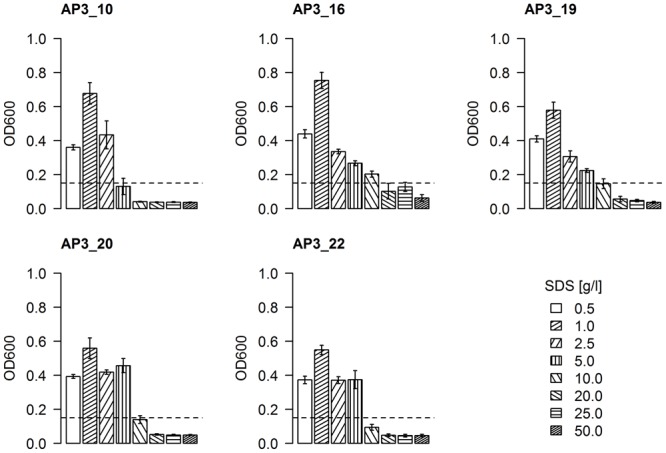
The effect of SDS concentration on the bacterial growth rates of selected strains reflected as optical density of cell cultures after 24 h of incubation. The values are means of the three replicates, and the error bars indicate the standard deviations. The dotted line represents the initial OD600 of each strain.

Having bacteria resistant to a high SDS concentration is an important characteristic for bioremediation. To assess the resistance of selected isolates to SDS, an increasing surfactant concentration was supplemented to cultures of each isolate in a range of 0.5–50 g/L. Bacterial growth, expressed as an increase of optical density (above the initial OD_600_ = 0.15), was found to be inversely proportional to increasing detergent concentration (**Figure 5**). All effective degrader isolates reached their highest optical density (OD_600_ = 0.55–0.75) in the presence of 1 g/L SDS, which could be interpreted as a sign of both a sufficient availability of the carbon source and the minimal negative effects of the detergent. The first SDS toxicity was observed in cultures supplemented by 2.5 g/L of SDS, where the optical density for all five isolates was similar or even below the levels observed for 0.5 g/L SDS supplemented samples. Presence of 5 g/L of detergent in medium had a significant toxic effects on the AP3_10 isolate, as the optical density of this culture did not change after 24 h (*p*-value = 0.76). Meanwhile, isolates AP3_16, AP3_19, AP3_20, and AP3_22 appeared to be relatively resistant in these conditions, as their cultures reached an optical density from 0.22 to 0.4 (*p*-value < 0.05). In the presence of 10 g/L of SDS, only isolate AP3_16 retained an ability to grow (*p*-value = 0.02). An intriguing behavior was observed for isolate AP3_16, which in contrast to the other isolates retained an OD_600_ of 0.12 in the presence of 20 and 25 g/L of detergent what was statistically not important change from initial OD_600_ = 0.15 (*p*-value = 0.45). Such result suggests that this strain maintained the ability at least to survive in the presence of high SDS concentrations, which to our knowledge has not been described before. However, detailed analysis of this phenomenon was not in the main scope of this report and will be investigated in future.

#### Chemotactic Response of the Isolates toward SDS (Drop Plate Assay)

**FIGURE 6:**
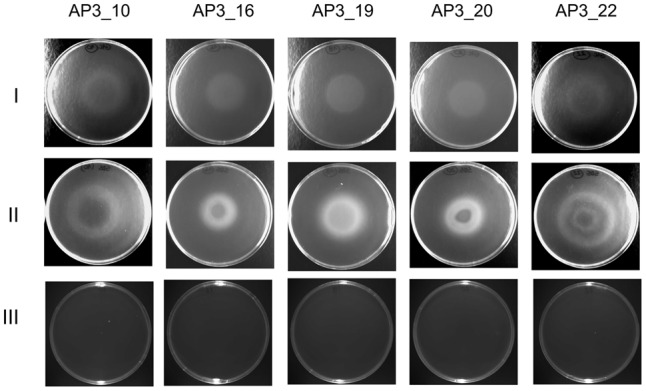
Drop plate assay for bacterial chemotaxis. Pictures of bacterial rings formed after 8 h incubation at 30°C. Rows represent selected strains’ chemotactic response toward glucose (I), SDS (II) and control plates without compound (III).

The chemotactic response of the five selected isolates toward SDS was studied by a drop plate assay, with glucose as a positive control. Growth rings indicating chemotaxis toward the given carbon source were observed after 8 h of incubation in the presence of both SDS and glucose (**Figure 6**). However, detailed analysis of growth patterns revealed differences between the analyzed isolates and carbon sources. On the positive control plates, each isolate migrated up to the center of the dish where the glucose was dropped, whereas on the plates with SDS only isolate AP3_19 exhibited migration up to the dish center. The remaining four isolates grew forming at least one ring of turbidity. For the sister taxa AP3_16, and AP3_19 isolates, which were able to survive at SDS concentrations exceeding 10 g/L, bacteria grew significantly closer to the plate center where the detergent was dropped. Whereas, growth rings formed by the SDS-vulnerable AP3_10 and AP3_22 isolates were notably wider, with no sign of bacterial presence in the plate center, where the SDS concentration was the highest. This observation indicate that the AP3_10 and AP3_22 isolates prefer lower detergent concentrations.

#### Analysis of SDS Degradation by Mass Spectrometry

For the five selected isolates, we employed mass spectrometry to confirm the SDS-degrading ability observed by colorimetric assay. For this purpose the selected isolates were grown in the same conditions as for colorimetric assay (liquid minimal medium supplemented with SDS or glucose as a control as the sole carbon source), and after 24 h the detergent concentration in the supernatant was confirmed by mass spectrometry. Moreover, the SPME-GC-MS method allowed for the identification of potential SDS degradation products.

##### LC-MS/MS quantitation of SDS concentration

The results obtained by LC-MS/MS analysis corresponded with the observations from the colorimetric assay (**Table 1**). Both methods confirmed a decrease in SDS concentration after incubation with each of the examined isolates. All of the isolates were able to degrade from 70 to 85% of the detergent during the 24 h of incubation in the liquid medium containing 1 g/L of SDS. Sodium dodecyl sulfate was not detected in the negative control (samples supplemented with glucose).

**Table 1:** Sodium dodecyl sulfate (SDS) concentration and OD_600_ in examined samples after 24 h of incubation.

Strain	SDS (g/l)		OD_600_
	Quantitation method		
	Colorimetric assay	LC-MS/MS	
AP3_10	0.263 ± 0.013	0.287 ± 0.012	1.015 ± 0.008
AP3_16	0.301 ± 0.002	0.333 ± 0.050	1.758 ± 0.013
AP3_19	0.232 ± 0.013	0.267 ± 0.015	1.605 ± 0.015
AP3_20	0.217 ± 0.012	0.223 ± 0.032	1.110 ± 0.016
AP3_22	0.203 ± 0.011	0.173 ± 0.021	1.554 ± 0.004
Control	1.096 ± 0.032	1.17 ± 0.115	0

*Initial SDS concentration in this experiment was 1 g/L SDS.*

##### SPME-GC-MS metabolites identification

SPME-GC-MS analysis was chosen for the identification of possible metabolites produced during SDS degradation. Clearly, 1-dodecanol seemed to be the most probable and simple compound belonging to this group. The GC-MS direct injection procedure of the supernatants or non-polar solvent extracts revealed thermal decomposition of SDS in the injection port of the chromatograph, indicating the presence of 1-dodecanol in pure SDS standard samples, thus making it impossible to distinguish the 1-dodecanol derived from biodegradation from that derived from thermal degradation of the analyzed samples. We therefore chose the SPME sample collecting technique, which was previously used with good effect for trace analysis in similar studies (Ouyang et al., 2011). In this technique, the volatile analyte is absorbed onto a coated fiber suspended over the sample in a closed vessel.

**FIGURE 7:**
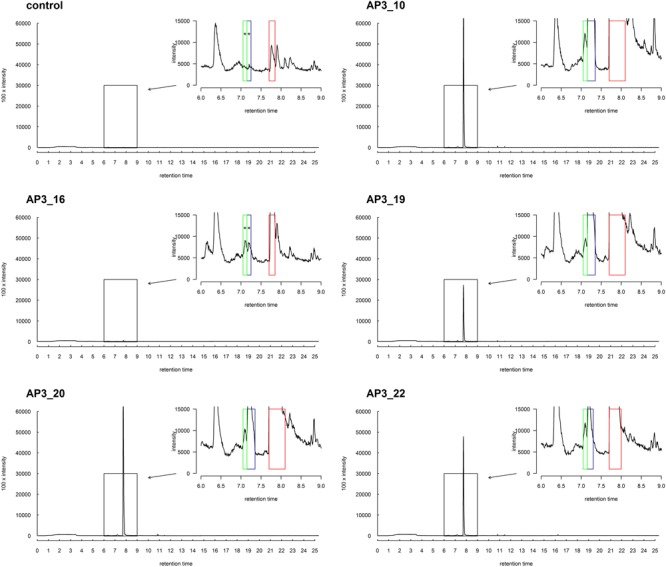
SPMS-GC-MS analysis of the SDS derivatives for selected strains after 24 h of incubation in minimal medium supplemented with 1 g/L SDS. The boxes indicate the peak positions for: 2-dodecanol and 3-dodecanol (green), dodecanal (blue), and dodecanol (red). MS-data (not-presented) from signals marked with asterisks were insufficient for certain confirmation of the presence of the compounds. However, the accordance of the retention times strongly suggests the presence of traces of these metabolites.

No degradation products were absorbed onto the fiber at the beginning of the bacterial growth, nor in the control samples, nor in any samples supplemented with glucose. However, after 24 h, a single chromatographic peak relating to 1-dodecanol was clearly observed in the GC-MS analysis in all bacterial cultures inoculated with SDS (**Figure 7**). This strongly suggests that 1-dodecanol is the main degradation product of SDS decomposition for the chosen bacteria isolates. We could also observe that the levels of identified 1-dodecanol varied among the isolates (see **Figure 7**), however, this observation needs further quantification. Spectra also showed presence of an aldehyde 1-dodecanal, another SDS metabolite, for which peak area was from 20 to 100 times smaller than the peak area observed for the main metabolite 1-dodecanol. What is interesting is that careful analysis of spectra for samples AP3_10, AP3_19, AP3_20, and AP3_22 showed traces of previously undescribed possible metabolites: 2-dodecanol and 3-dodecanol. In contrast, none of these by-products was identified in the AP3_16 sample – exhibiting the smallest peak related to 1-dodecanol, suggesting that this isolate has the fastest metabolism among the identified isolates.

#### Detection of Enzymes Engaged in SDS Degradation – Native PAGE Zymography

**FIGURE 8:**
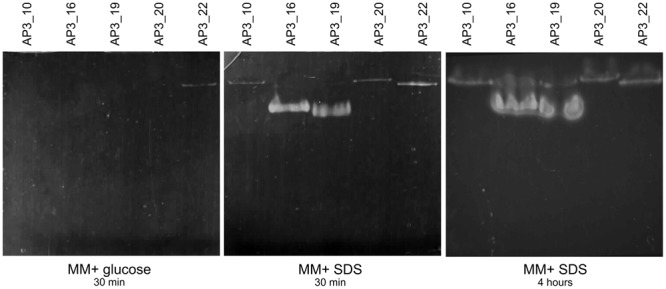
Native PAGE zymography of the five best SDS-degrading strains.

SPME-GC-MS analysis revealed 1-dodecanol as the main by-product formed in the presence of bacteria, which suggests that bacterial alkyl sulfatases could be involved in the metabolism of the detergent. This hypothesis was tested with native PAGE zymography. Native PAGE electrophoresis of crude cell extracts from isolates incubated in the presence of SDS showed presence of visible bands, which indicates that enzymes present in all five tested isolates had the ability for SDS desulfurylation, and thus exhibited alkyl sulfatase activity. Three isolates, AP3_10, AP3_20, and AP3_22, harbored one band on zymography, suggesting the presence of only one enzyme with alkyl sulfatase activity toward SDS (see **Figure 8**). Whereas for AP3_16 and AP3_19, which are phylogenetically distinct from the others, two bands were visible on zymography, suggesting the presence of two different alkyl sulfatases. Presence of almost all of the identified bands was correlated with the presence of the SDS in the medium. Except AP3_22 sample no activity was detected in cell extracts from isolates cultivated on glucose. Activity observed in the AP3_22 sample was expressed constitutively, and the band appeared on zymography in cell extracts originating from both glucose and SDS cultures.

## Discussion

### Properties of the Isolates with Regard to SDS Degradation

Although all of the isolates described here originated from one soil sample, biochemical tests showed significant differences in their SDS-degrading properties. This diversity was assessed by examination of differences in the halo formed around isolated colonies grown on basal medium supplemented with SDS as the sole carbon source. Further experiments confirmed the abilities of isolates for SDS decomposition and allowed for the identification of the 36 most promising SDS degraders. From this number, AP3_22 turned out to be the most effective isolate, as it decomposed 81% of 1 g/L of SDS within 8 h.

### Analysis of the Microbial Taxonomic Biodiversity of the Isolated SDS-Degrading Bacteria and the Soil Sample

In next step, we wanted to place the isolates that efficiently degrade one of the most popular detergent on the globe into a phylogenetic context. For this purpose, at first we analyzed microbial taxonomic biodiversity of AP3 sample to build the broad picture of the environment inhabited by the isolates. At the same time, we performed a detailed taxonomic classification of the selected isolates to see which microorganisms culturable in laboratory may be involved in sodium dodecyl sulfate decomposition.

Peaty soil analyzed here was inhabited by a diverse microbial consortium grouped in 45 different phyla. This richness was present despite the fact that the soil was regularly contaminated with chemicals like pesticides or detergents. *Proteobacteria* was the most abundant phylum (33.52%) in the AP3 soil sample, which stands in accordance to previous reports, where *Proteobacteria* were found to be the most common phylum for many soil habitats, having a large impact on global carbon, nitrogen, and sulfur cycling (Liles et al., 2003; Tringe et al., 2005; Janssen, 2006; Fulthorpe et al., 2008). Within this phylum *Alphaproteobacteria*, one of the most abundant bacterial class in soil environments (Spain et al., 2009), was represented in the AP3 sample, consisting of 10.60% of the sequences. The *Gammaproteobacteria* consisting of *Pseudomonas, Stenotrophomonas*, and *Sphingomonas*, known from their ability to degrade a wide range of chemical compounds of human origin (Spain et al., 2009), were represented by only 0.89% of sequences in the AP3 sample.

Interestingly, the phylum *Parcubacteria* seemed to be one of the major phyla in the AP3 soil sample (16.04%). These uncultured microorganisms are rarely reported in soil or terrestrial environments, and are usually identified in marine and freshwater sediments, mesophilic sulfur springs or lately, in alpine permafrost (Elshahed et al., 2005; Briée et al., 2007; Frey et al., 2016). Microorganisms from this phylum were characterized as having a reduced genome and presumably mutualistic, commensal, or parasitic lifestyles. But until now, there was no evidence showing that microorganisms from phylum *Parcubacteria* were found in environments contaminated with detergents or other xenobiotics. On the generic level, the AP3 sample showed no dominant group of microorganisms. However, many of them like *Crenothrix* or *Geobacter* (Stoecker et al., 2006; Butler et al., 2007; Feld et al., 2015) had been previously isolated from similar soil contaminated environments.

Bioinformatic analysis of the V3–V4 16S rRNA amplicon of the 36 selected SDS degrading isolates revealed that all of the microorganisms presented here belong to *Pseudomonas*. Surprisingly only 0.58% of the sequences present in the total DNA were assigned to this genus. This suggests that *Pseudomonas* could be one of the culturable groups of microorganisms inhabiting analyzed peaty soil, and play an important role in naturally occurring bioremediation of SDS.

Further sequencing of the 16S rRNA amplicons of the SDS fast degraders showed that the selected isolates were closely related to *P. jessenii* subgroup. Detailed taxonomic assignment of above isolates has yet to be confirmed; nevertheless, it needs to be emphasized that this is the first time that microorganisms that cluster in the same clade as *P. jessenii, P. moorei, P. vancouverensis, P. reinekei, P. koreensis*, and *P. moraviensis* have been shown to have SDS-degrading capabilities. This group may also include *P. umsongensis*, and *P. mohnii*, and is distinct from other *Pseudomonas* (**Figure 4**).

This finding sheds more light on the environmental distribution of detergent-degrading *Pseudomonas*, which up to now have been mainly identified and isolated from samples such as soil, active sludge, and aqueous systems. Moreover, it enlarges the group of known *Pseudomonas* representatives, such as *P. aeruginosa, P. alcaligenes, P. beteli, P. mendocina, P. otitidis, P. pseudoalcaligenes, P. putida, P. plecoglossicida* S5, *P. stutzeri* and other *Pseudomonas* spp., known to have SDS decomposition abilities (Payne and Feisal, 1963; Kahnert and Kertesz, 2000; Hagelueken et al., 2006; Klebensberger et al., 2006; Jovčić et al., 2009; Chaturvedi and Kumar, 2011b; Yeldho et al., 2011; John et al., 2015).

The previously described *P. aeruginosa* MTCC 10311, isolated from surfactant-contaminated soil, was able to degrade 96% of 1.5 g/L SDS in 48 h (Ambily and Jisha, 2012), whereas *P. aeruginosa* S7 isolate by Yeldho from contaminated soil degraded 70% of 1 g/L SDS during 24 h (Yeldho et al., 2011). There were reported several *Pseudomonas* spp. strains isolated from water ponds in India (Chaturvedi and Kumar, 2010, 2011a) having similar degree of SDS decomposition to the isolates described here.

There were reported several *Pseudomonas* spp. strains isolated from water ponds in India (Chaturvedi and Kumar, 2010, 2011a) were reported to have a similar degree of SDS decomposition to the isolates described here. The *P. putida* strain SDS3 and *P. otitidis* strain NN1 identified by Chaturvedi and Kumar degraded 1 g/L of SDS almost completely within 12 h. There are also examples of bacteria taxonomically distant to *Pseudomonas* like *A. johnsonii* and *P. beteli*, isolated by Hosseini from active sludge, degrading 96.4 and 97.2% of 0.522 g/L SDS, respectively, within 10 days of incubation in minimal medium (Hosseini et al., 2007). Another example was the *K. oxytoca* isolated from polluted water samples utilized almost 80% of 2 g/L SDS during 4 days of incubation (Shukor et al., 2009). Due to the fact that studies described in the literature have not been performed under similar conditions, it is difficult to compare directly the SDS-degrading capabilities of the *Pseudomonas* isolates reported here to microorganisms previously described by others. Nevertheless, a literature search showed that isolates from AP3 soil sample, especially AP3_22 belong to the group of the most effective SDS decomposers isolated from terrestrial environments.

### Resistance of the Isolates to High SDS Concentrations

Resistance to the toxic effect of high detergent concentrations is another feature that helps to identify potentially useful detergent-degrading microorganisms. It is known that an SDS concentration exceeding 2 g/L kills most bacteria by stripping away the lipopolysaccharide outer layer and denaturing membrane proteins (Odahara, 2004). Our results suggest that two out of five of the identified SDS-degrading isolates have the ability to survive in the presence of 10 g/L of SDS. This experiment also showed an intriguing quality of AP3_16. This isolate, retained initial cell culture optical density and cell lysis was not observed in the presence of 20 and 25 g/L of detergent. Survival in the presence of such high SDS concentrations has not, to our knowledge, been described before. Moreover, observations from drop plate assays seem to correlate with these results, showing the influence of different SDS concentration on the growth of isolates. Bacteria tolerating SDS concentrations exceeding 10 g/L grew significantly closer to the plate center where the detergent was dropped, whereas rings formed by isolates vulnerable to high detergent concentrations were notably wider.

A literature search for the maximum limit of SDS tolerated by *Pseudomonas* showed that *Pseudomonas* sp. DRY15, isolated from Antarctic soil, was completely inhibited at 5 g/L SDS (Halmi et al., 2013). Whereas *P. aeruginosa* MTCC 10311, identified by Ambily and Jisha (2012), could survive up to 20 g/L of SDS.

### Analysis of Possible SDS Metabolism By-Products

The degree of SDS degradation measured with the colorimetric assay was confirmed by the LC-MS/MS method. More importantly, for all five efficient SDS degraders, mass spectrometry (SPME-GC-MS) was employed to analyze the detergent degradation products.

The LC-MS/MS results showed that the colorimetric Stains-All assay, similarly to MBAS (**m**ethylene **b**lue **a**ctive **s**ubstances assay) method (Shahbazi et al., 2013), may be considered as an inexpensive, fast, and accurate method for sodium dodecyl sulfate quantitation, allowing for easy adaptation to high throughput screening protocols.

The SPME-GC-MS analysis confirmed that in the selected five isolates, dodecanol is the main SDS degradation product. However, in the case of isolates: AP3_10, AP3_19, AP3_20, and AP3_22, the SPME-GC-MS method allowed for the identification of low levels of dodecanal, 2-dodecanol, and 3-dodecanol, which could be regarded as SDS degradation by-products. In contrast to previous studies (Thomas and White, 1989; Ambily and Jisha, 2014), we were unable to detect traces of dodecanoic or decanoic acid. This could be explained by the fact that isolates described here were effectively utilizing SDS metabolites as a source of energy or integrating them into cellular membranes. According to Thomas and White (1989), experiments using radiolabeled [1-14C] SDS showed that its degradation is initiated by the sulfatase-mediated hydrolysis of ester to alcohol. Dodecanol is then oxidized to dodecanal, and then further on to dodecanoic acid, which is either elongated to long-chain fatty acids and incorporated into membrane phospholipids or used as an energy source via the tricarboxylic acid cycle.

GC-MS analysis showed that despite the isolates exhibiting a similar degree of SDS bioconversion to dodecanol (measured as a decrease in the substrate concentration), they differed in the levels of this alcohol present in the culture medium after 24 h. Moreover, the ratio of identified dodecanal:dodecanol never exceeded 5% in any case. The observed differences in the detected dodecanol levels very likely reflect differences in bacterial metabolism.

Interestingly, in the case of the AP3_16 isolate, which had extremely high tolerance to SDS (25 g/L), we were unable to detect any derivatives, except low amounts of dodecanol. This could be explained by the fact, that the isolate probably possessing very active enzymatic machinery involved in the subsequent biodegradation steps or an efficient transport system for the derivatives within the bacterial cell. This is probably why even traces of dodecanal, 2-dodecanol, or 3-dodecanol could not be detected in the culture medium after 24 h of AP3_16 incubation with SDS.

On the other hand, in samples from the AP3_10 isolate, one of the less tolerant toward SDS (growing only up to 2.5 g/L), we could observe all of the aforementioned compounds with relatively high amounts of dodecanol.

### Identification of Alkylsulfatase Activity in the Isolates

Native PAGE zymography revealed that all of the selected isolates possessed alkylsulfatase-like activity. Three isolates, AP3_10, AP3_20, and AP3_22, produced a single band that could be the result of alkylsulfatase activity, whereas for isolates AP3_16 and AP3_19 two bands were observed. Intriguingly, there was no correlation between the number of identified potential alkyl sulfatases and the SDS degradation products observed with SPME-GC-MS. Several previously published reports suggest that microorganisms involved in SDS degradation could produce single (Hagelueken et al., 2006; Chaturvedi and Kumar, 2010, 2011b; Jovcic et al., 2010) or multiple alkylsulfatases, such as *Pseudomonas* C12B – five enzymes or *P. putida* FLA – six enzymes (Lillis et al., 1983). Our results also suggest that the alkyl sulfatase activity in four out of the five tested isolates is regulated by the presence of SDS in the culture media, similar to the results obtained by, for instance, Chaturvedi and Kumar (2010). However, the AP3_22 isolate harbored a constitutively expressed alkyl sulfatase, such as the P1 sulfatase from *Pseudomonas* C12B (Toesch et al., 2014).

## Conclusion

In this study, we used peaty soil sampled from a biological wastewater treatment plant to isolate and identify 36 *Pseudomonas* isolates exhibiting the ability to use SDS as their sole carbon source. Five isolates, having close taxonomic relationships to *P. jessenii, P. moorei, P. vancouverensis, P. reinekei, P. koreensis*, and *P. moraviensis* appeared to be the most efficient SDS degraders, decomposing from 80 to 100% of the SDS present at an initial concentration 1 g/L within 24 h. Despite their similarity, all isolates belonging to the *P. jessenii* subgroup exhibited significant differences in the dynamics of their SDS degradation, chemotaxis toward SDS on a plate test, and resistance to high detergent concentrations (see **Table 2**). The detergent metabolism among these five effectively degrading isolates was reflected by the byproducts observed with mass spectrometry, where beside the dominating and well-known 1-dodecanol, we observed traces of dodecanal, 2-dodecanol, and 3-dodecanol, but no dodecanoic acid. The discrepancy between the isolates identified here was confirmed by a zymography test that strongly suggested that the closely related microorganisms isolated from one soil sample employed more than one alkyl sulfatase for SDS desulfurylation, which is considered as the initial step for detergent decomposition. The above observations indicate that isolates described here exhibit exceptional capabilities for detergent decomposition and have developed very efficient mechanisms to survive and flourish in very high SDS concentrations. In order to better describe the above phenomena, we plan to extend the analysis at the genomic and metabolomic levels. Analysis of the complete genome sequence of these microorganisms could allow us to identify alkyl sulfatase enzymes, which are responsible for SDS decomposition. Moreover, genome analysis will be helpful to decipher mechanisms that allow those microorganisms to survive in presence of SDS exceeding 10 g/L. Future discoveries will open a way to develop biochemical tools to manage SDS in either natural or industrial environments.

**Table 2:** Summary of the characteristic of the fast degrader strains.

Strain	AP3_10	AP3_16	AP3_19	AP3_20	AP3_22
Closest relative according to EzBioCloud database	*Pseudomonas jessenii* CIP 105274	*Pseudomonas jessenii* CIP 105274	*Pseudomonas mohnii* Ipa2	*Pseudomonas jessenii* CIP 105274	*Pseudomonas jessenii* CIP 105274
Chemotaxis toward SDS	(+) One ring observed	(+) One ring observed with almost no empty space inside	(+) One ring observed with no empty space inside	(+) One ring observed	(+) Two rings observed
Zymography assay	One band induced by SDS	Two bands induced by SDS	Two bands induced by SDS	One band induced by SDS	One band constitutive
Product of SDS degradation	Mainly dodecanol; dodecanal and traces of 2-dodecanol, 3-dodecanol	Dodecanol	Mainly dodecanol; dodecanal and traces of 2-dodecanol, 3-dodecanol	Mainly dodecanol; dodecanal and traces of 2-dodecanol, 3-dodecanol	Mainly dodecanol; dodecanal and traces of 2-dodecanol, 3-dodecanol
Maximum acceptable SDS concentration	2.5 g/L	10 g/L	5 g/L	5 g/L	5 g/L

## Author Contributions

AS and LL conceived and directed the studies. MK participated in bacteria isolation from soil. Bacterial phenotype analysis, DNA isolation, DNA libraries preparation, amplicon sequencing and all bioinformatics analysis were performed by EF. GS, MS, and WD designed and performed the LC-MS/MS and SPME-GC-MS analysis with MS data interpretation. The manuscript was written by EF, consulted and corrected by AS, LL, and AD. Funding for this work was provided by LL and AD. All the authors read and approved the final manuscript.

## Conflict of Interest Statement

The authors declare that the research was conducted in the absence of any commercial or financial relationships that could be construed as a potential conflict of interest. The reviewer CP and handling Editor declared their shared affiliation, and the handling Editor states that the process nevertheless met the standards of a fair and objective review.
