# Supplementary material for: Isolation and Characterization of Pseudomonas spp. Strains That Efficiently Decompose Sodium Dodecyl Sulfate
Source: Front Microbiol. 2017 Nov 7;8:1872. doi: 10.3389/fmicb.2017.01872 (PMC5681903; doi:10.3389/fmicb.2017.01872)
Supplement: Supplementary file 1 [file Table_1.DOCX]

| Strain | SDS concentration [%] at different time points | | | | | | OD600 at different time points * | | | | | |
| --- | --- | --- | --- | --- | --- | --- | --- | --- | --- | --- | --- | --- |
|  | 0 h | 2 h | 4 h | 6 h | 8 h | 24 h | 0 h | 2 h | 4 h | 6 h | 8 h | 24 h |
| **AP3_15** | 119.5±19.7 | 109.8±5.6 | 114.3±5.8 | 111.7±8.8 | 109.1±7.6 | 99.4±4.6 | 0.058 | 0.034 | 0.020 | 0.015 | 0.014 | 0.007 |
| **AP3_25** | 98.5±6.0 | 106.3±4.4 | 96.9±4.6 | 96.4±5.7 | 115.6±5.3 | 97.7±2.6 | 0.070 | 0.038 | 0.038 | 0.040 | 0.040 | 0.017 |
| **AP3_13** | 103.9±12.7 | 103.2±3.8 | 103.8±3.6 | 92.3±9.1 | 91.0±3.2 | 93.6±1.5 | 0.040 | 0.034 | 0.050 | 0.023 | 0.116 | 0.209 |
| **AP3_27** | 93.5±6.0 | 85.4±1.3 | 101.1±0.8 | 85.6±0.5 | 94.1±5.2 | 90.9±2.7 | 0.114 | 0.067 | 0.100 | 0.157 | 0.213 | 0.408 |
| **AP3_33** | 104.6±7.2 | 103.9±1.9 | 100.5±1.6 | 99.6±0.9 | 99.4±1.7 | 90.3±1.6 | 0.104 | 0.081 | 0.106 | 0.165 | 0.229 | 0.368 |
| **AP3_21** | 105.2±3.7 | 99.8±0.7 | 92.8±2.0 | 89.0±2.8 | 103.2±0.3 | 90.3±1.0 | 0.043 | 0.016 | 0.019 | 0.026 | 0.031 | 0.151 |
| **AP3_26** | 95.2±3.1 | 92.5±4.0 | 89.3±4.3 | 93.3±4.2 | 85.6±6.3 | 82.3±1.4 | 0.069 | 0.048 | 0.087 | 0.125 | 0.178 | 1.144 |
| **AP3_14** | 94.8±0.6 | 90.0±5.1 | 105.4±1.3 | 96.3±2.6 | 96.7±3.6 | 82.0±2.1 | 0.106 | 0.077 | 0.078 | 0.086 | 0.104 | 0.487 |
| **AP3_18** | 104.8±3.1 | 107.1±11.5 | 100.5±8.9 | 105.9±5.8 | 91.5±5.3 | 80.9±2.1 | 0.076 | 0.082 | 0.158 | 0.229 | 0.276 | 0.631 |
| **AP3_6** | 103.3±1.8 | 94.7±0.9 | 86.0±1.3 | 85.7±3.4 | 85.1±0.1 | 80.3±2.2 | 0.051 | 0.073 | 0.142 | 0.076 | 0.057 | 0.032 |
| **AP3_30** | 102.2±3.1 | 97.3±5.6 | 96.3±4.0 | 97.2±4.7 | 101.9±6.8 | 80.3±1.4 | 0.057 | 0.066 | 0.094 | 0.169 | 0.266 | 0.452 |
| **AP3_8** | 91.7±4.8 | 84.0±2.4 | 86.4±3.2 | 84.7±7.7 | 84.9±6.8 | 79.2±3.8 | 0.088 | 0.068 | 0.127 | 0.165 | 0.249 | 0.283 |
| **AP3_12** | 110.9±5.7 | 110.4±2.6 | 102.8±1.1 | 97.5±2.0 | 95.5±3.8 | 78.8±1.5 | 0.116 | 0.114 | 0.143 | 0.231 | 0.289 | 0.423 |
| **AP3_2** | 104.9±2 | 101.1±1.2 | 100.3±3.5 | 98.0±7.4 | 103.3±3.9 | 73.2±6.0 | 0.04 | 0.015 | 0.019 | 0.031 | 0.041 | 0.445 |
| **AP3_35** | 91.8±3.8 | 96.5±2.6 | 87.4±4.2 | 89.8±8.6 | 92.3±4.6 | 72.4±3.6 | 0.057 | 0.063 | 0.105 | 0.186 | 0.314 | 1.167 |
| **AP3_1** | 110.5±2.7 | 109.6±6.7 | 99.3±2.5 | 101.2±2.0 | 99.5±3.1 | 71.7±2.5 | 0.056 | 0.03 | 0.038 | 0.069 | 0.067 | 0.529 |
| **AP3_28** | 99.9±1.6 | 95.8±1.7 | 96.6±6.0 | 88.9±1.6 | 80.7±1.5 | 55.9±1.1 | 0.119 | 0.135 | 0.020 | 0.324 | 0.334 | 0.557 |
| **AP3_9** | 102.9±11.1 | 100.3±2.4 | 97.8±4.4 | 93.0±4.2 | 88.1±0.5 | 54.4±4.6 | 0.110 | 0.111 | 0.160 | 0.305 | 0.419 | 0.693 |
| **AP3_32** | 97.6±4.5 | 103±3.6 | 92.6±3.1 | 93.9±4.4 | 97.6±3.7 | 53.4±1.4 | 0.037 | 0.034 | 0.067 | 0.124 | 0.180 | 0.806 |
| **AP3_3** | 106.6±1.9 | 103.7±3.8 | 99.3±3.1 | 89.4±2.8 | 90.1±5.6 | 50.4±0.9 | 0.043 | 0.049 | 0.104 | 0.157 | 0.236 | 0.922 |
| **AP3_29** | 106.4±3.1 | 100.7±1.9 | 91.9±1.8 | 88.1±5.8 | 88.2±1.4 | 50.0±1.2 | 0.086 | 0.093 | 0.113 | 0.156 | 0.223 | 1.124 |
| **AP3_36** | 89.8±3.2 | 101.6±2.3 | 60.9±3.1 | 42.9±3.0 | 49.9±1.3 | 47.9±3.4 | 0.149 | 0.142 | 0.252 | 0.461 | 0.281 | 0.853 |
| **AP3_34** | 105.5±2.7 | 103.5±0.9 | 94.6±1.8 | 83.9±7.5 | 86.4±1.7 | 46.4±1.1 | 0.062 | 0.043 | 0.073 | 0.132 | 0.194 | 0.819 |
| **AP3_4** | 100.8±0.7 | 92.0±3.0 | 97.6±1.6 | 84.4±0.6 | 87.8±1.1 | 43.1±1.1 | 0.138 | 0.14 | 0.193 | 0.333 | 0.475 | 1.185 |
| **AP3_31** | 98.1±3.2 | 98.8±2.6 | 99.5±6.4 | 91.5±3.2 | 90.4±5.8 | 42.2±1.0 | 0.135 | 0.129 | 0.168 | 0.272 | 0.350 | 1.28 |
| **AP3_23** | 98.6±3.7 | 89.9±2.0 | 104.1±1.8 | 90.9±1.3 | 84.0±1.9 | 41.3±0.3 | 0.104 | 0.088 | 0.123 | 0.259 | 0.355 | 1.075 |
| **AP3_11** | 100.4±1.7 | 93.6±4.8 | 98.8±1.3 | 88.5±1.6 | 86.1±2.9 | 40.9±0.6 | 0.129 | 0.111 | 0.136 | 0.238 | 0.434 | 1.133 |
| **AP3_7** | 107.9±1.4 | 100.2±3.0 | 103.6±1.1 | 95.4±12 | 99.9±2.2 | 39.0±0.6 | 0.068 | 0.075 | 0.09 | 0.141 | 0.245 | 1.096 |
| **AP3_5** | 111.4±3.2 | 105.8±4.6 | 102.7±3.4 | 98.2±0.9 | 91.4±2.2 | 37.5±0.5 | 0.095 | 0.041 | 0.076 | 0.119 | 0.172 | 1.202 |
| **AP3_24** | 86.3±11.9 | 85.9±5.4 | 101.1±0.8 | 88.2±0.7 | 95.2±4.9 | 31.8±1.3 | 0.103 | 0.067 | 0.103 | 0.132 | 0.181 | 1.26 |
| **AP3_17** | 101.1±1.8 | 97.8±3.2 | 104.3±5.2 | 99.2±3.6 | 100.1±0.5 | 31.1±0.6 | 0.109 | 0.083 | 0.111 | 0.149 | 0.217 | 1.443 |
| **AP3_10** | 102.3±1.9 | 97.0±1.4 | 95.2±5.5 | 88.6±1.8 | 89.3±5.5 | 25.9±0.3 | 0.107 | 0.097 | 0.119 | 0.229 | 0.368 | 1.102 |
| **AP3_20** | 98.4±2.4 | 92.9±2.8 | 103.1±3.6 | 91.9±1.6 | 93.9±2.6 | 20.6±1.1 | 0.106 | 0.090 | 0.127 | 0.193 | 0.329 | 1.153 |
| **AP3_16** | 106.4±3.2 | 98.3±0.5 | 97.5±4.3 | 85.3±1.6 | 71.8±0.7 | 15.2±0.4 | 0.132 | 0.125 | 0.206 | 0.389 | 0.582 | 1.579 |
| **AP3_19** | 94.8±0.7 | 96.7±3.0 | 101.7±4.1 | 76.9±1.7 | 76.6±1.6 | 14.9±0.8 | 0.100 | 0.112 | 0.248 | 0.445 | 0.610 | 1.439 |
| **AP3_22** | 97.2±2.1 | 90.1±1.2 | 92.1±4.9 | 50.9±0.6 | 18.9±3.4 | 00.8±1.2 | 0.136 | 0.110 | 0.196 | 0.312 | 0.473 | 1.210 |

**Table S1.** Detailed results of the SDS biodegradation screening assay. Isolates are sorted and grouped by the degradation rate at the end of the experiment into: non-, slow, medium and fast degraders.

* sd <0.01 at each measurement

**
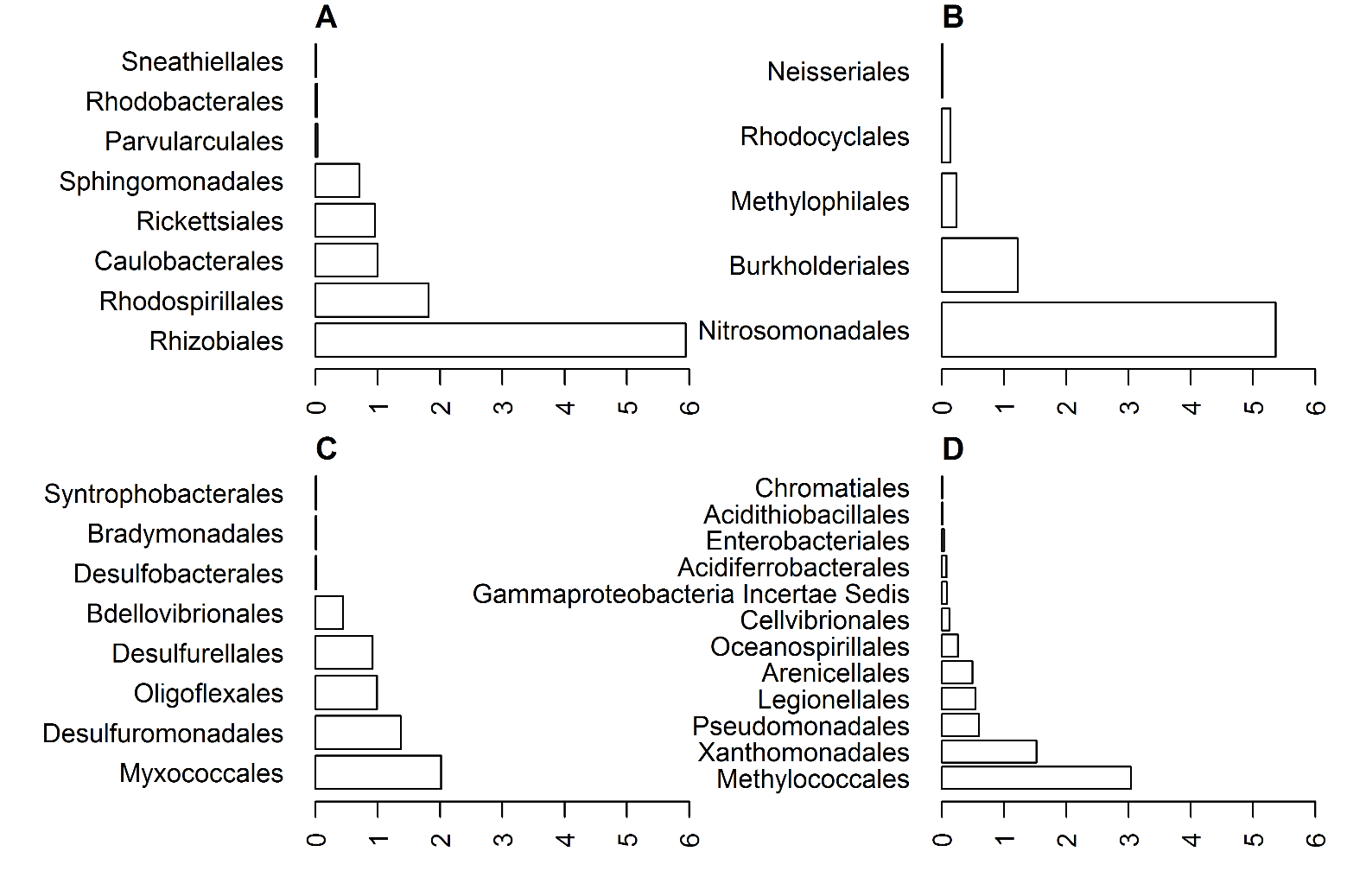
**

**Fig S1.** AP3MET soil sample bacterial community composition at different taxonomic ranks (at least 0.01% abundance): A- order level inside *Alphaproteobacteria* class, B- order level inside *Betaproteobacteria* class, C - order level inside *Deltaproteobacteria* class, D - order level inside *Gammaproteobacteria* class.
